# Room‐Temperature Skyrmionic Synapse in 2D Ferromagnet Fe_3_GaTe_2_ Operating via Collective Spin Texture Transformation

**DOI:** 10.1002/adma.202520288

**Published:** 2026-05-20

**Authors:** Jixiang Huang, Tongji Zhu, PeiYu Cai, Xiaoming Ma, Zhen Wang, Ruifu Zhang, Yi Hao, Jingdi Lu, Liubing He, Jun Xu, Yuejie Zhang, Wanjun Jiang, Jing Tao, Shiming Lei, Pavel Parchinskiy, Jing Teng, Lingfei Wang, Wei Niu, Elton J.G. Santos, Xiaoqian Zhang, Peng Li

**Affiliations:** ^1^ School of Microelectronics University of Science and Technology of China Hefei Anhui China; ^2^ Institute for Condensed Matter and Complex Systems School of Physics and Astronomy The University of Edinburgh Edinburgh UK; ^3^ College of Integrated Circuits and Optoelectronic Chips Shenzhen Technology University Shenzhen Guangdong China; ^4^ Department of Physics University of Science and Technology of China Hefei Anhui China; ^5^ School of Integrated Circuits Huazhong University of Science and Technology Wuhan Hubei China; ^6^ Hefei National Research Center for Physical Sciences at Microscale University of Science and Technology of China Hefei China; ^7^ State Key Laboratory of Low‐Dimensional Quantum Physics and Department of Physics Tsinghua University Beijing China; ^8^ Frontier Science Center for Quantum Information Tsinghua University Beijing China; ^9^ Department of Physics Hong Kong University of Science and Technology Hong Kong China; ^10^ Department of Semiconductor and Polymer Physics National University of Uzbekistan Tashkent Uzbekistan; ^11^ Beijing National Laboratory for Condensed Matter Physics Institute of Physics Chinese Academy of Sciences Beijing China; ^12^ School of Physical Sciences University of Chinese Academy of Sciences Beijing China; ^13^ New Energy Technology Engineering Laboratory of Jiangsu Province & School of Science Nanjing University of Posts and Telecommunications Nanjing China; ^14^ Higgs Centre for Theoretical Physics The University of Edinburgh Edinburgh UK; ^15^ Donostia International Physics Center (DIPC) Donostia‐San Sebastián Spain; ^16^ Key Laboratory of Quantum Materials and Devices of Ministry of Education School of Physics Southeast University Nanjing China

**Keywords:** artificial synapse, multiply‐accumulate, room‐temperature 2D magnet, skyrmion

## Abstract

Magnetic skyrmions, as topologically protected spin textures, hold great potential for energy‐efficient neuromorphic systems. While artificial synapses have been demonstrated in magnetic multilayers by electrically controlling skyrmion populations, their probabilistic nucleation severely limits reliability. The recent emergence of 2D van der Waals magnets, with their inherent tunability and novel spintronic phenomena, offers a promising platform to overcome these challenges. Here, we demonstrate an artificial synaptic device in 2D ferromagnet Fe_3_GaTe_2_, operating on the fundamentally different principle of a deterministic and collective spin texture transformation from a skyrmion‐lattice to a stripe‐domain state. This transformation yields a linear, reproducible modulation of the anomalous Hall resistance. The slope of this linear response, defined as the synaptic weight, is effectively tuned by varying the pulse width, thereby enabling multi‐weight functionality and multiply‐accumulate operations. Projected scaling of the device reduces the single‐operation energy consumption to 0.66 pJ, a level comparable to state‐of‐the‐art memristor technologies (e.g., resistive random‐access memory and phase‐change memory). Furthermore, a hardware‐informed quantized neural network based on this synapse achieves a high recognition accuracy (∼96.1%) in handwritten‐digit recognition. Our findings establish a robust pathway for creating large‐scale and energy‐efficient neuromorphic systems based on the collective dynamics of Fe_3_GaTe_2_ spin textures at room temperature.

## Graphical abstract

We demonstrate a neuromorphic synapse in 2D Fe_3_GaTe_2_ flakes. The device operates via a current‐driven transformation from a skyrmion‐lattice to a stripe‐domain state, yielding a linear anomalous Hall resistance response with a tunable slope to enable multiply‐accumulate operations. Simulations confirm its viability in artificial neural networks. This work broadens the application prospects for skyrmions and 2D magnets.

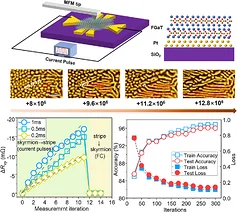

## 1 Introduction

Magnetic skyrmions are topologically nontrivial spin configurations characterized by a vortex‐like arrangement of magnetic moments [1]. Their stabilization arises from the competition among Dzyaloshinskii–Moriya interaction (DMI), dipolar interactions, magnetic anisotropy energy, and exchange interaction [2]. Initially discovered in the bulk chiral magnet MnSi [3], skyrmions have since been realized in magnetic thin films [4], magnetic multilayers [5], engineered heterostructures [6, 7], etc. Over the past years, significant advances have been achieved in skyrmion nucleation [6, 8], detection [8, 9, 10, 11, 12], annihilation [13], and manipulation [14, 15]. Among various manipulation approaches, electrical driving based on spin‐transfer torque (STT) and spin–orbit torque (SOT) is of particular importance for skyrmion‐based devices by offering fast, energy‐efficient, and scalable control of skyrmions [16, 17, 18]. Especially, owing to their nanoscale dimensions and topological protection, skyrmions can be driven with ultralow current densities and ultra‐high velocity [18, 19, 20], enabling the construction of high‐performance, low‐power spintronic devices, such as logic [21], memory [22], and unconventional computing [23, 24].

Among the many potential applications for skyrmionics, neuromorphic computing has recently received widespread attention. This brain‐inspired computing paradigm aims to address the “memory wall” limitation [25] of conventional von Neumann architectures by co‐locating memory and logic units. A fundamental building block of such a system is the artificial synapse, whose primary functions are to retain and update non‐volatile synaptic weights, thereby enabling efficient multiply‐accumulate (MAC) operations (*Y* = Σ*W*_i_·*X*_i_) on incoming signals [26]. For hardware implementation, magnetic skyrmions have emerged as highly promising candidates for realizing artificial synapses due to their intrinsic non‐volatility and analog programmability [27]. Indeed, recent work has successfully demonstrated the plasticity of artificial synapses and performed MAC operations by electrically controlling the number of skyrmions [28, 29]. However, the creation of individual skyrmions is often a fundamentally probabilistic process. This stochasticity can introduce errors during weight updates and degrade the precision of MAC operations. While accuracy can be improved by accumulating a large number of operations, this comes at the cost of higher energy consumption [29]. Therefore, developing a new scheme to achieve deterministic skyrmion‐based synaptic behavior is essential for building high‐precision, energy‐efficient neuromorphic hardware.

Remarkably, the emergence of 2D van der Waals (vdW) magnets offers an outstanding platform to address this challenge, owing to their pristine interfaces and extensive tunability [30, 31]. Among these materials, the recently reported room‐temperature 2D ferromagnet Fe_3_GaTe_2_ (FGaT) is especially noteworthy. This novel magnet exhibits a Curie temperature (*T*_C_) well above 350 K and strong perpendicular magnetic anisotropy (PMA), even in exfoliated thin flakes [32]. Moreover, it has been proposed that Fe vacancies inherent to non‐stoichiometric FGaT crystals can break the bulk inversion symmetry, giving rise to the DMI necessary to stabilize skyrmions [33, 34]. Consequently, Lorentz transmission electron microscopy characterizations have established the existence of skyrmions in FGaT [33, 34, 35, 36] and demonstrated the manipulation of skyrmions using a variety of external stimuli, including magnetic fields [36], intercalation [37], electric currents [38, 39], and strain [40, 41]. Meanwhile, prior works have realized artificial synaptic devices in FGaT using field‐free SOT switching and electric‐field control of magnetization [42, 43], indicating that the FGaT platform emerges as a highly promising candidate for realizing synaptic devices. However, achieving deterministic skyrmion control for enabling the required low‐power and high‐reliability synaptic operation, as a critical challenge, has remained unrealized until now.

Here, we report a novel manipulation protocol based on the current‐driven, collective, and deterministic transformation of a skyrmion‐lattice into a stripe‐domain state in 2D ferromagnetic FGaT flakes and implement it as a functional synaptic element. The protocol leverages the progressive evolution of spin textures triggered by current pulses, operating on a principle distinct from conventional methods that rely on the stochastic nucleation of individual skyrmions. This transformation generates a deterministic and robust change in the anomalous Hall resistance (AHR), enabling electrical detection of the spin texture evolution. Crucially, a linear relationship is established between the AHR reduction and the number of injected pulses, where the slope—corresponding to the synaptic weight—can be effectively modulated by the pulse width, enabling multi‐level synaptic functionality. Furthermore, the power consumption analysis projects that for a device scaled to a 180 nm technology node, the energy consumption for a single synaptic operation can be as low as 0.66 pJ. Based on this electrically‐tunable synaptic device, a hardware‐informed quantized artificial neural network (QANN) is constructed, achieving a recognition accuracy exceeding 96% in a handwritten‐digit recognition task. These findings establish a complete and achievable pathway from controlled skyrmion manipulation in FGaT to functional neuromorphic synaptic devices, underscoring the significant potential of FGaT‐based skyrmion dynamics for next‐generation neuromorphic computing applications.

## 2 Experimental Results

### 2.1 Crystal Structure and Magnetic Domain Characterizations of Fe_3_GaTe_2_

**FIGURE 1:**
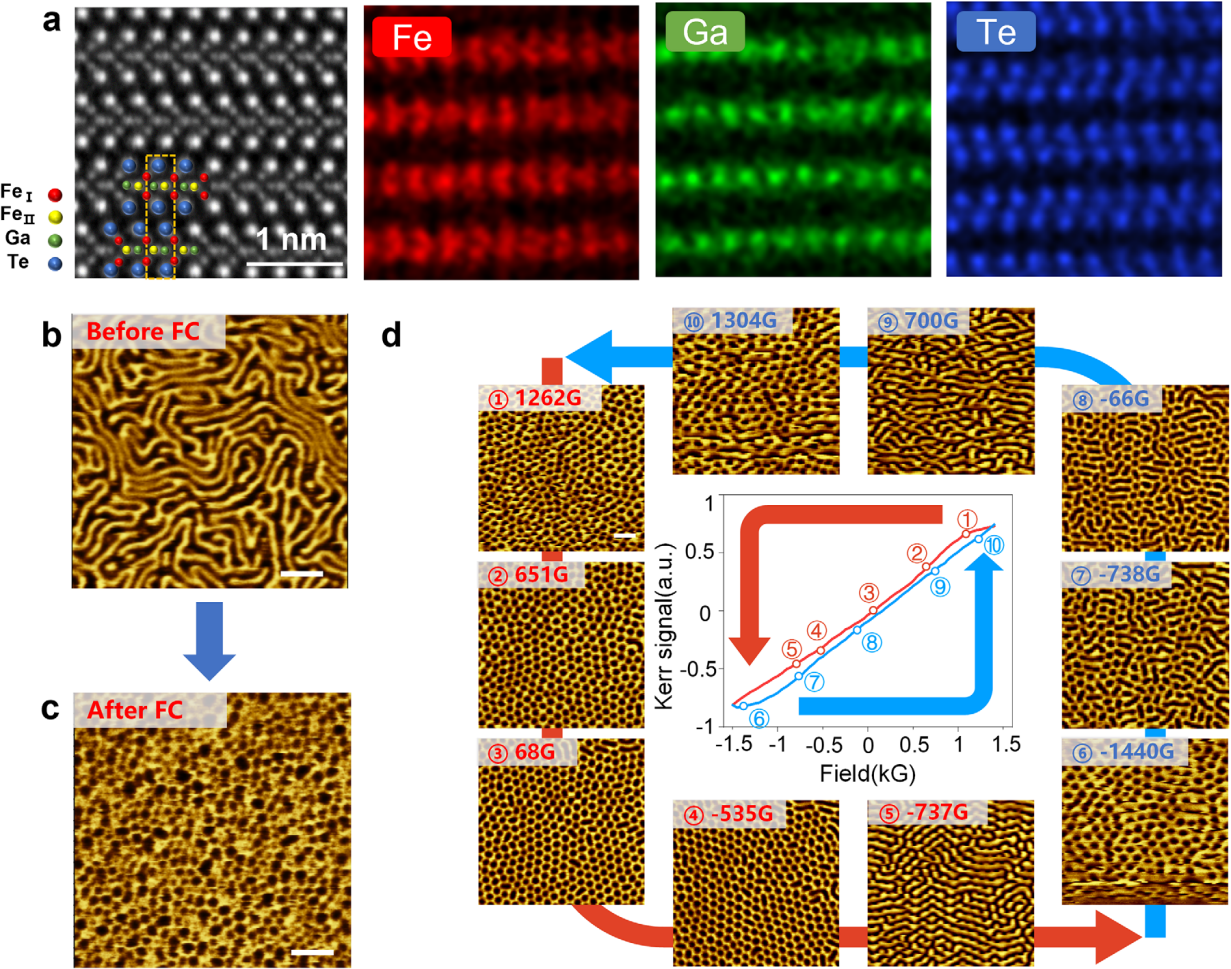
Structure and magnetic domain characterization of FGaT. (a) HAADF‐STEM image and EDX maps of FGaT viewed along the [100] direction. The inset shows the schematic of the atomic structure. The red, yellow, green, and blue atoms represent Fe_I_, Fe_II_, Ga, and Te atoms, respectively. (b,c) MFM images of an exfoliated FGaT flake showing (b) the initial stripe‐domain ground state and (c) the resulting skyrmion lattice after a FC procedure. (d) Sequential MFM images capturing the subsequent field‐driven evolution of the skyrmion lattice at room temperature, showing the reversible transformation between skyrmion‐lattice and stripe‐domain states. The scale bar for all MFM images is 1 µm.

High‐quality FGaT single crystals synthesized via a Bridgman‐like method (see Experimental Section) were characterized using high‐angle annular dark‐field scanning transmission electron microscopy (HAADF‐STEM). Figure 1a displays a high‐resolution cross‐sectional HAADF‐STEM image of the FGaT lamella, captured along the [100] orientation. The atomic arrangement exhibits a pristine periodic lattice, where the quasi‐2D layers are separated by well‐defined vdW gaps. As illustrated in the corresponding atomic model, each Fe‐Ga slab is sandwiched between two Te atomic layers, and adjacent Te layers are separated by a distinct vdW gap. Within the slabs, Fe atoms occupy two inequivalent Wyckoff sites (Fe_I_ and Fe_II_), forming a Te‐Fe_I_‐Fe_II_/Ga‐Fe_I_‐Te stacking sequence along the *c*‐axis [33, 34]. Complementary energy‐dispersive X‐ray (EDX) spectroscopy mapping (Figure 1a, right panels) confirms the uniform spatial distribution of Fe, Ga, and Te elements throughout the flake [44].

Having established the high quality of FGaT, magnetic force microscopy (MFM) was adopted to visualize the topological spin textures. As exhibited in Figure 1b, a 62‐nm‐thick FGaT flake spontaneously forms a labyrinthine stripe domain configuration at zero magnetic field. To generate skyrmions, a field‐cooling (FC) protocol was performed as previously reported [36], wherein the flake was heated above its *T*_C_ and subsequently cooled back to room‐temperature under an out‐of‐plane (OOP) magnetic field of approximately 400 G. This procedure effectively transforms the stripe domains into a densely packed skyrmion lattice, as shown in Figure 1c. Although Fe_3_GaTe_2_ is nominally centrosymmetric in its ideal crystal structure, real samples are known to exhibit Fe‐site disorder between the Fe_I_ and Fe_II_ sublattices, as reported in previous structural studies [33, 34]. Such an antisite disorder breaks the inversion symmetry, which can generate a finite DMI. Moreover, it is evidenced that the magnetic domain configuration in FGaT flakes is strongly influenced by competing interactions between thickness‐dependent dipole–dipole interactions, sample oxidation, and PMA (Figures S1–S3). Crucially, systematic thickness studies reveal that ordered stripe domains, which are essential precursors for skyrmion formation, only stabilize above a critical thickness threshold, while unprotected samples show degradation of these magnetic textures due to ambient oxidation. These findings collectively demonstrate that achieving controllable skyrmion states requires OOP field cooling, optimal thickness selection, and effective encapsulation strategies.

To probe the stability and dynamics of these magnetic skyrmions, the evolution of these magnetic skyrmions as a function of the magnetic field was systematically examined. After initializing the sample into the skyrmion‐lattice state, the external OOP magnetic field was swept, with the resulting spin textures recorded by MFM (Figure 1d). Starting from 1262 G (state 1), the skyrmion lattice remains stable as the field is first reduced to 651 G (state 2) and further down to 68 G (state 3). As the field is decreased to zero and then reversed, the system undergoes a sequential transformation from skyrmions to stripe domains (state 5), and then to skyrmions again under a large negative magnetic field (state 6). A subsequent reverse sweep from ‐1440 G back toward positive fields reproduces this complex evolution. This demonstrates that the spin textures in FGaT are interconvertible, with the domain configuration being a function of both the instantaneous applied magnetic field and its application history, revealing a significant memory effect [45]. Notably, when an OOP magnetic field exceeding the saturation field of FGaT is applied, all skyrmions are annihilated. Upon removal of the field, the system relaxes into a labyrinth‐domain configuration. Furthermore, field‐driven evolution initiated from stripe domains does not exhibit a skyrmion‐stripe transformation (Figure S4). This field‐history‐dependent control over distinct topological spin textures underscores the potential of FGaT as a promising material platform for developing neuromorphic devices [23].

### 2.2 Deterministic Current‐Induced Skyrmion‐Stripe Transformation

**FIGURE 2:**
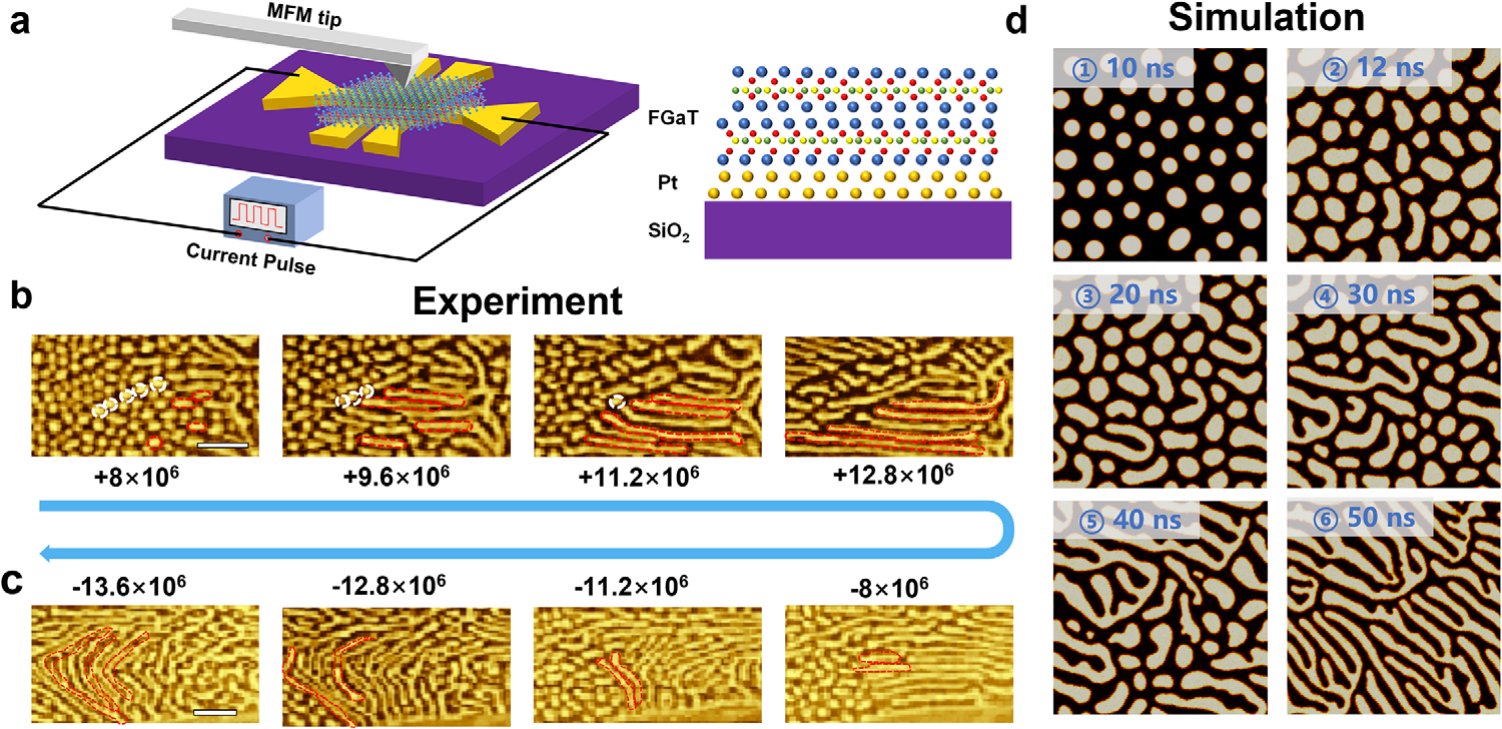
Current‐driven skyrmion‐stripe transformation in FGaT. (a) Schematic of the experimental test device, with a cross‐sectional view shown on the right. (b,c) MFM images showing the spin texture evolution under a train of (b) positive and (c) negative current pulses. The applied current density is noted below each image, and arrows indicate the sequence of pulses. (d) Micromagnetic simulation snapshots of skyrmion‐stripe transition in a 1200 × 1200 × 60 nm^3^ geometry with 7 × 10^6^ A/cm^2^ current density, the capture time is labeled in each diagram. The scale bars in (b,c) are 1 µm. All the MFM measurements were performed at room temperature.

Guided by the principle that direct electrical control is essential for scalable spintronics, we focused on current‐induced skyrmion dynamics in FGaT. Accordingly, heterostructure devices were fabricated by transferring exfoliated FGaT flakes onto pre‐patterned Pt electrodes (Figure 2a). Current pulses with an amplitude on the order of 10^7^ A/cm^2^ were applied to the Pt/FGaT device, followed by MFM imaging to track the magnetic domain evolution. As depicted in Figure 2b, applying positive current pulses causes skyrmions at the edge of the current path to elongate and transform into stripe domains. With continued pulsing, the domain walls of these stripes propagate inward. This domain wall propagation (tracked by the red dashed lines), exerts pressure on the interior skyrmions, compressing and ultimately annihilating them (indicated by white dashed circles). To overcome pinning effects [46] caused by material inhomogeneity and to ensure consistent domain wall motion, the pulse amplitude was incrementally increased during the experiment. This process continued until the skyrmions were completely replaced by the stripe‐domain phase. Furthermore, reversing the polarity of the applied current pulses produced a corresponding reversal of the domain wall motion direction (Figure 2c). This current‐direction‐dependent response excludes Joule heating as the dominant driving mechanism, given that it scales with *I* ^2^ and is inherently direction‐independent. In contrast, the linear dependence of spin torques on the direction of the injected current pulses establishes them as the dominant driving mechanism for the observed domain wall propagation [38, 39].

To gain deeper insights into the observed dynamics, we performed micromagnetic simulations initialized by mimicking the experimental FC protocol. Starting from a random paramagnetic state, the system was cooled to 4 K under an 80 mT OOP field and subsequently equilibrated at 300 K to incorporate realistic thermal fluctuations (see Experimental Section for details).

As shown in Figure 2d, a stable skyrmion lattice configuration is established at *t* = 10 ns (state 1). Upon removal of the external field and simultaneous application of a spin‐polarized current, the system undergoes rapid relaxation due to the vanishing Zeeman energy, settling into a quasi‐stable skyrmion lattice state by 12 ns (state 2). With continuous current injection, the simulation reproduces the experimentally observed evolution: skyrmions gradually elongate into stripe domains, and the current‐driven domain wall propagation leads to the annihilation of adjacent skyrmions. By *t* = 50 ns, the spin texture has fully transformed into a stripe domain phase (state 5). This transition is further quantified by the evolution of the topological charge (Figure S8), which decreases from ∼ −50 (skyrmion lattice) to ∼ −5 (stripe domains), confirming the skyrmion annihilation and stripe expansion.

Furthermore, an analysis of the individual energy terms (Figure S9) reveals that the transformation is governed by a competition between the thermodynamic driving force of the demagnetization field and the energy barriers imposed by exchange and anisotropy interactions. Specifically, the stripe‐domain state acts as the thermodynamic ground state in the zero‐field regime by minimizing stray fields. Conversely, the elongation of skyrmions increases the total domain wall length and tilts the magnetization, imposing energy penalties visible in the rising of the exchange and anisotropy contributions, which are followed by the decrease of the demagnetization energy. Crucially, the net increase in total energy during the pulse confirms that the SOT and STT currents actively inject energy to overcome these barriers [47, 48]. Consequently, the mechanism is an energy‐injection‐assisted relaxation, where the current pushes the system over the topological barrier, allowing it to relax downhill in the demagnetization energy landscape toward the stable stripe phase. Moreover, the simulation results reveal that the transition from skyrmions to stripe domains is accompanied by a decrease in magnetization (*M*_z_, Figure S10). This finding provides a clear physical basis for the electrical readout of the skyrmion‐stripe transformation [29].

### 2.3 Electrical Readout: AHR as a Probe for Spin Texture Evolution

**FIGURE 3:**
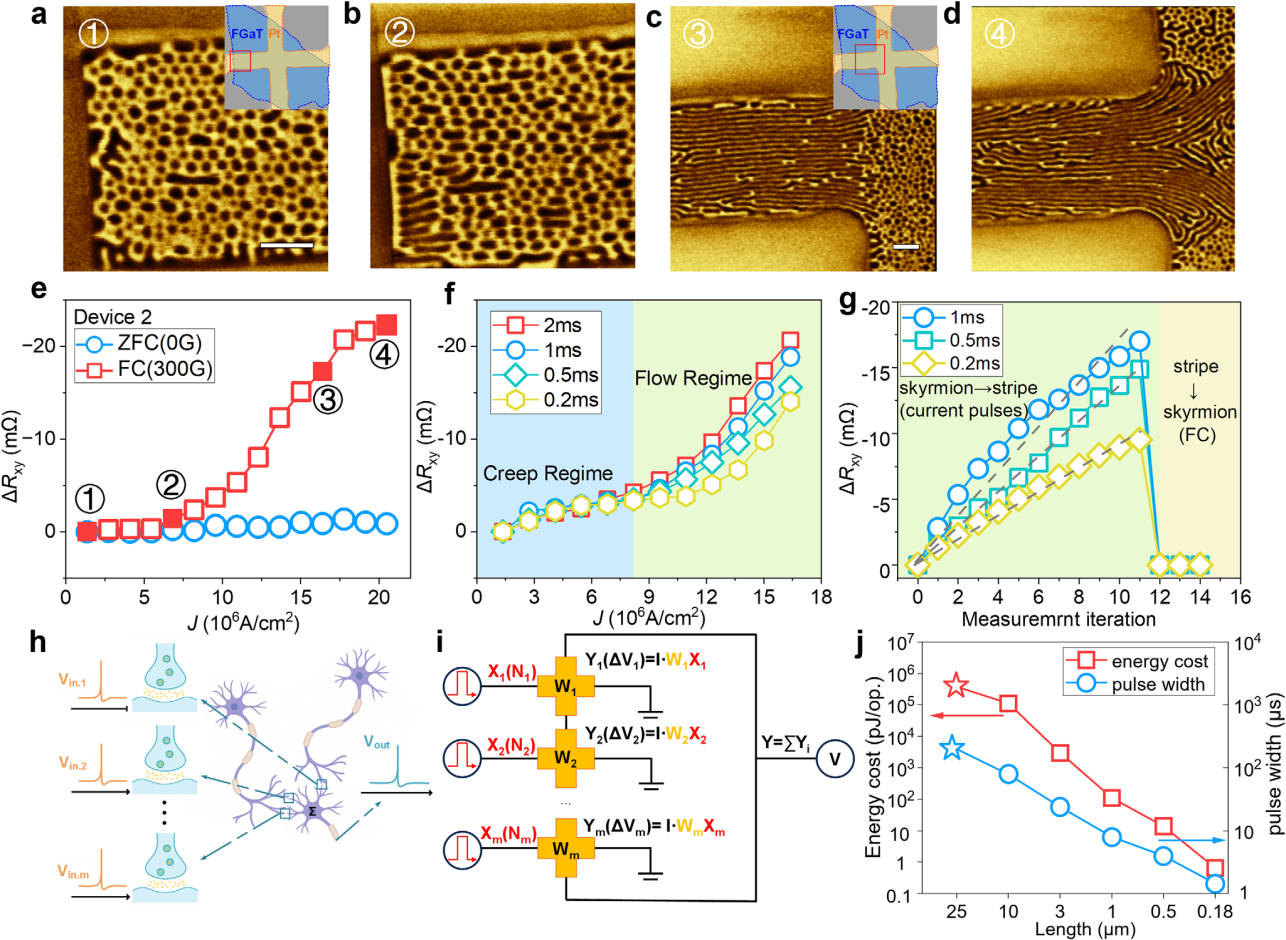
Electrical readout of FGaT skyrmion evolution. (a–d) Evolution of the device's MFM images with applied current pulses. The insets in (a,c) are photos of the device under test, where the red squares indicate the region of interest in (a–d), respectively. The scale bar is 1 µm. (e) Change of the AHR in the device under applied current pulses. Red and blue points represent FC and ZFC conditions, respectively. The numbered data points (1–4) correspond directly to the magnetic states shown in panels (a–d). f) Dependence of the AHR change (Δ*R*_xy_) on current pulse amplitude for various pulse widths, showing the creep and flow regimes. (g) Accumulated change in Δ*R*_xy_ as a function of the number of applied pulses in the flow regime. The linear fits are shown as gray dashed lines. (h,i) Schematic illustrating the principles of MAC operation in (h) biological synapses and (i) our proposed skyrmion‐based synaptic device. (j) Projected scaling of energy cost per synaptic operation and required pulse width with device miniaturization. The plot shows the calculated energy per operation (red squares, left axis) and the necessary pulse width (blue circles, right axis) as a function of the device track length. The projection assumes a constant device aspect ratio of 1:1. The stars indicate the values for the 25 µm × 5 µm device used in this study, which serves as the baseline for the scaling analysis.

Having demonstrated current‐driven dynamics of FGaT skyrmions, we now turn to quantifying their electrical signature by monitoring the evolution of the AHR in order to construct a stable synaptic device (Figure 3). A modified FGaT/Pt heterostructure device fabricated by etching is employed to conduct the AHR test (see Experimental Setup). Figure 3a–d present MFM images that illustrate the evolution of the spin texture as a series of current pulses are applied to the device, which was initially prepared in a skyrmion state via FC. The images reveal a gradual, collective transformation from a dense skyrmion lattice (Figure 3a) to a state dominated by stripe domains (Figure 3d). This physical transformation is quantitatively captured by the AHR response, as shown in Figure 3e. When the device is in the initial skyrmion state, applying successive current pulses induces a significant and monotonic decrease in Δ*R*_xy_ (the change in AHR, marked by red squares). The data points labeled 1–4 on the graph directly correspond to the respective spin textures observed in the MFM images (Figure 3a–d), confirming that the change in electrical resistance is a direct measure of the collective spin texture transformation. In sharp contrast, when the device starts from a stripe‐domain configuration (zero‐field‐cooling, ZFC), identical current pulses produce a negligible change in Δ*R*_xy_(marked by blue circles, corresponding MFM images are listed in Figure S12). This comparison unequivocally establishes that the change in Hall resistance serves as a robust and high‐fidelity electrical probe for the transition of skyrmion lattice to stripe domain.

To further understand the dynamics, we systematically investigated the evolution of Δ*R*_xy_ as a function of current amplitude and pulse width (Figure 3f). Two distinct dynamic regimes (marked by blue and green) are identified. At low current amplitudes, the system operates in a creep regime, which is consistent with previous observation [49]. Under these circumstances, domain wall motion is thermally assisted over a random pinning landscape, resulting in a slow and gradual reduction in Δ*R*_xy_. Above a threshold current, the system transitions into a flow regime, where spin torques overcome the pinning potential, leading to faster domain wall propagation and a much more rapid decrease in resistance. Notably, for a given current amplitude, longer pulse widths result in a larger change in Δ*R*_xy_, as they allow for greater domain wall displacement.

Crucially, the controlled and cumulative nature of this transition is particularly evident in the flow regime, as shown in Figure 3g. Here, the observed linear relationship between Δ*R*_xy_ and the number of applied pulses (*N*_i_) was engineered through a tailored current drive scheme: incrementing the density of the current pulses from 9.6 to 13.6 × 10^6^ A/cm^2^ with a step of 4 × 10^5^ A/cm^2^ to overcome local pinning effects, ensuring reproducible domain transitions from skyrmion to stripe. The slope of the linear fits (gray dashed lines) represents the resistance change per pulse, and by varying the pulse width from 0.2 to 1 ms, this slope can be effectively tuned.

To verify the reproducibility of the AHR response, we fabricated devices with varying geometric dimensions and repeated the measurements. The results confirm that the behaviors observed in Figure 3f,g can be consistently reproduced across different devices. This finding reaffirms the feasibility of electrically detecting the FGaT spin texture transition via the AHR signature (Figures S14 and S15).

## 3 Discussions

### 3.1 FGaT Skyrmionic Synapses Construction and Performance Benchmark

This electrically tunable, linear response provides a direct physical realization of a synaptic function, inspired by the weighted sum operations found in biological neural networks (Figure 3h). In such a system, a neuron receives input signals (action potentials), and its synapses transmit this information with specific weights (*W*_i_) by releasing a corresponding amount of neurotransmitters, which then accumulate at the postsynaptic neuron to perform a weighted summation [50, 51]. As shown in Figure 3i, our proposed scheme directly emulates this biological process. The number of input pulses (*N*_i_) acts as the input signal (*X*_i_), while the rate of resistance change per pulse (Δ*R*_xy_/*N*_i_) corresponds to the synaptic weight (*W*_i_). The total accumulated resistance change (*W*_i_ × *X*_i_) can be readily converted to an output Hall voltage (Δ*V*_xy_) by injecting a constant read current (*Y*_i_ = *I* × *W*_i_ × *X*_i_). By connecting multiple such devices in series, their individual output Hall voltages naturally sum due to the additive nature of voltage. The total measured voltage thus represents the final weighted sum (*Y* = Σ*Y*_i_), effectively realizing the full MAC functionality that forms the foundation of hardware‐based neuromorphic computing.

It is worth noting that our device operates based on a collective spin texture transformation within FGaT, which offers a distinct advantage in operational reliability. Unlike devices that rely on the stochastic nucleation of individual skyrmions, the collective behavior in our device provides a more deterministic pathway for weight modulation, ensuring higher precision for MAC operations. Additionally, the way of combining spin information with electrical transport demonstrates that FGaT‐based neuromorphic computing is Complementary Metal Oxide Semiconductor (CMOS)‐compatible. The primary trade‐offs for this mechanism are the relatively high energy consumption and slower operating speed, as indicated by the baseline measurements in Figure 3j. However, these limitations are primarily due to the geometric dimensions of the current device (an active area of 25 × 5 µm^2^), which were chosen to ensure a detectable AHR signal but also necessitate longer pulse widths to achieve complete transformation. These challenges can be significantly mitigated through device scaling and improved readout methods. For instance, if a more advanced magnetic tunnel junction (MTJ) were used for readout [52], the device could be substantially miniaturized. As the projection in Figure 3j illustrates, scaling the device down to 0.18 × 0.18 µm^2^ can reduce the required pulse width to 1.44 µs and lower the single‐operation energy consumption to 0.66 pJ. This projected energy efficiency is comparable to that of current resistive random‐access memory (RRAM) [53] and phase‐change memory (PCM) [54] technologies, and represents a significant improvement over CMOS‐based synapses that utilize static random‐access memory (SRAM) and a capacitor [55, 56]. Recent advances in wafer‐scale FGaT film growth [57] will significantly improve device interface quality and reduce domain wall pinning effects, offering a clear pathway toward further reducing the device's energy consumption. Furthermore, the development of FGaT‐based MTJ [58] is crucial for realizing highly integrated device arrays. As indicated in our analysis (Figure S18), such arrays are projected to exhibit a superior integration density compared to CMOS‐based synapses [55, 56].

Besides, multi‐cycle write‐erase tests were performed and demonstrate that the weight update operation in our skyrmionic synaptic devices is stable and reproducible. (Figure S16). We attribute this robustness to the collective and deterministic nature of the spin texture transitions in FGaT. Moreover, our results indicate that the device response exhibits negligible drift over time. Consequently, our devices satisfy the critical endurance and retention requirements for general synaptic applications [59]. These analyses confirm that synaptic devices based on FGaT skyrmion‐stripe transformation have a clear and viable pathway toward achieving the high precision, low power, and high speed required for practical neuromorphic computing hardware.

### 3.2 Neuromorphic Computing Simulation with FGaT Skyrmionic Synapses

**FIGURE 4:**
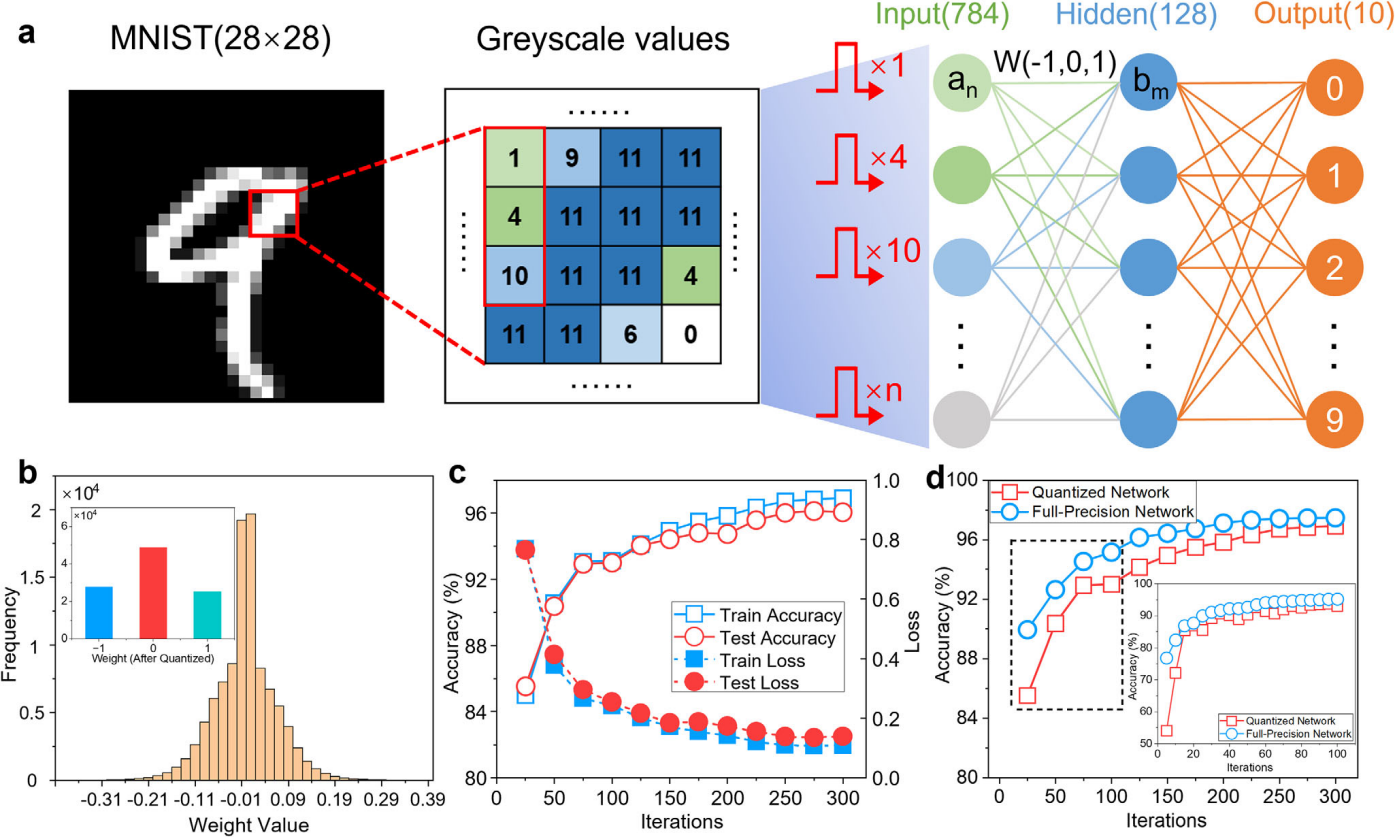
Neuromorphic computing simulation based on FGaT skyrmionic synapse. (a) Schematic of the pattern recognition task and the hardware mapping strategy. An input image from the MNIST dataset (left, digit ‘9’) has its pixel grayscale values (middle, showing the region in the red box) encoded into the number of applied current pulses. This input is then processed by a three‐layer artificial neural network (right; 784 × 128 × 10 architecture), where the synaptic weights are quantized to three discrete levels: ‐1, 0, + 1. (b) Distribution of the full‐precision synaptic weights after network training. The inset shows the final weight distribution after quantification. (c) Recognition accuracy and loss curves of the QANN model, where red and blue represent the training and testing datasets, respectively. (d) Comparison of recognition accuracy between the quantized model and the full‐precision network, which serves as a baseline. The data within the black box is further detailed in the inset.

To further explore the potential of the demonstrated synaptic characteristics for implementing neuromorphic systems, the physical response of the FGaT‐based device is mapped onto the MAC operations of a neural network and subsequently validated through system‐level simulations. For practical application, a QANN was designed to perform a handwritten digit recognition task on the standard Modified National Institute of Standards and Technology (MNIST) dataset. As shown in Figure 4a, each 28 × 28 pixel input image is first flattened into a 784‐element vector, corresponding to the 784 neurons of the input layer of the network [60]. The grayscale value of each pixel (typically ranging from 0 to 255) is first normalized to a unit interval and then linearly scaled by a factor of 11. The resulting value is subsequently rounded to the nearest integer, yielding 12 discrete levels ranging from 0 to 11. Crucially, each of these integer values corresponds directly to the number of physical current pulses to be applied to a synaptic device in the hardware implementation. This input quantization scheme ensures that the image data is encoded in a format directly compatible with the physical operating mechanism of our FGaT skyrmionic synapse.

Subsequently, we defined the three‐layer network architecture shown in the right panel of Figure 4a and employed a specific Quantization‐Aware Training (QAT) strategy to train the synaptic weights [61]. Within this framework, we use a standard Kaiming uniform distribution [62] to initialize the weights and then employ a dynamic ternarization method to quantize the weights [63]. For each forward propagation, these full‐precision weights were dynamically first quantized to three discrete target values: ‐1, 0, + 1 (see Experimental Section), which correspond to the distinct slopes measured in Figure 3g. The forward computation is then performed using these quantized weights. Following each forward propagation, gradients were calculated in the backward pass to generate updated full‐precision weights. This training scheme is designed to directly mirror the physical operation of our device. In a hardware implementation, the three quantized weights correspond to three distinct current pulse widths. Therefore, updating a weight does not require a separate operation, and it is simply achieved by selecting the appropriate pulse width for the input pulse train in the next computational cycle. This approach greatly reduces the time and energy budget typically associated with weight adaptation in memristive systems. The final weight distribution after training is presented in Figure 4b, where the inset confirms that the QAT process successfully trained the weights to cluster into the three target discrete states, validating the effectiveness of our strategy.

The performance of the trained QANN is summarized in the accuracy and loss curves presented in Figure 4c. After 300 training iterations, the network achieves a training accuracy of 96.91% and a testing accuracy of 96.06%. Concurrently, the training and test loss values converge to low values of 0.11–0.14, respectively. This confirms the reliability and generalization capability of our hardware‐inspired QANN model. In Figure 4d, the recognition accuracy of our quantized network is compared with that of a full‐precision network, which serves as a performance baseline. It is obvious that our quantized model can achieve an accuracy comparable to that of the 97.46% of the full‐precision network. The inset in Figure 4d provides a detailed comparison of the initial training stage, revealing that the full‐precision network converges to a high accuracy more rapidly. This suggests that pursuing a greater number of weight states in future devices is a key direction for optimization. Recent advances in wafer‐scale FGaT film growth [57] and the development of MTJ‐based readout schemes [58] will allow for the pursuit of more weight states and the construction of device arrays in the future. These developments are poised to significantly enhance the application prospects of devices based on FGaT spin texture transformations for neuromorphic computing.

## 4 Summary and Outlook

In conclusion, this work demonstrates a new type of artificial synapse by harnessing the current‐driven, deterministic and collective transformation of skyrmions into stripe domains at room temperature in 2D ferromagnet FGaT. This operational paradigm, based on a deterministic spin texture transformation rather than a stochastic skyrmion nucleation, offers a substantial advantage in computational precision. The process yields a robust electrical signature in the AHR, exhibiting a tunable, multi‐level memristive response analogous to a synaptic weight. Leveraging this functionality, we designed and validated a hardware‐informed quantized artificial neural network, which achieved a high recognition accuracy of over 96% on the MNIST dataset. This study is the first to propose and implement the function of a synaptic device based on a collective spin texture transformation in a 2D magnet at room temperature, validating its significant potential for neural network applications. Based on the guidelines included in our work, near‐future advancements in wafer‐scale film growth and integration of MTJ‐based crossbar arrays in CMOS‐friendly setups are expected to sizably reduce power consumption to the sub‐pJ regime. These developments will facilitate the application of room‐temperature FGaT‐based synapses in large‐scale neural networks in practical computing tasks.

## 5 Experimental Section

### 5.1 Crystal Synthesis

FGaT single crystals were synthesized by the Bridgman‐like method. High‐purity elemental Fe (99.98%), Ga (99.999%), and Te (99.999%) were mixed and placed into a quartz ampoule. The ampoule was subsequently evacuated and sealed. The entire assembly was heated in a box furnace to 1150°C and held at this temperature for 50 h to ensure a homogeneous melt. Following this, the furnace was slowly cooled to 500°C over a period of 100 h, after which it was shut off to allow the sample to cool naturally to room temperature. Single crystals of FGaT were subsequently harvested from the surface of the solidified mixture.

### 5.2 Device Fabrication

Two types of Pt/FGaT heterostructure devices were fabricated in this study. For the device primarily used for the MFM tests shown in Figure 2 (Device 1), a 5 nm Pt film was first deposited on a Si/SiO_2_ substrate via magnetron sputtering at a power of 50 W in a 3 mT Ar atmosphere. The Pt layer was then patterned into electrodes using a Suss MABA6 Mask aligner, followed by reactive ion etching (RIE). Finally, an FGaT flake, obtained by mechanical exfoliation, was transferred onto the pre‐patterned Pt electrodes. For the devices used for electrical transport and corresponding MFM measurements (Devices 2–3), the fabrication process began with the mechanical exfoliation of FGaT flakes onto a Si/SiO_2_ substrate inside a N_2_‐filled glovebox to prevent degradation. Prior to Pt deposition, the substrate surface was cleaned for 25 s using a 100 W RF bias in a 5 mT Ar atmosphere. A 10 nm Pt film was then deposited using the same parameters. The resulting heterostructure was subsequently patterned using the same UV lithography and RIE processes.

### 5.3 MFM Imaging

MFM measurements were employed to visualize the magnetic domain structures at room temperature. Field‐dependent skyrmion evolution experiments were performed using an Oxford Instruments Asylum Research MFP‐3D atomic force microscope. The current‐driven skyrmion evolution experiments were conducted on a Bruker Dimension Icon atomic force microscope (AFM). For experiments involving current pulses, an initial MFM scan was taken to image the domain state. The MFM tip was then fully withdrawn from the surface, a current pulse was applied to the device, and the tip was subsequently re‐engaged for a new scan. This procedure was used to prevent any potential artifacts from the current influencing the magnetic tip.

### 5.4 Micromagnetic Simulation

Micromagnetic simulations were performed using the open‐source, GPU‐accelerated MUMAX3 package [64]. To ensure direct comparability with experimental data, material parameters were derived from measurements at room temperature. Detailed material parameters are: exchange stiffness *A*_ex_ = 7.5 × 10^−12^ J/m, first order uniaxial anisotropy *K*_u1_ = 2.0 × 10^5^ J/m^3^, and saturation magnetization *M*_sat_ = 3.76 × 10^5^ A/m [37]. Besides, interfacial DMI strength D = 1.07 × 10^−3^ J/m^2^, Landau‐Lifshitz damping constant alpha = 0.02 [36].

The simulation grid was dimensioned into a grid of 300 × 300 ×15 cells, with each sized at 4 × 4 × 4 nm^3^. The discretized cell size was set below the calculated exchange length and anisotropy length, ensuring numerical validity. Periodic boundary conditions (PBCs) were applied in the x and y directions to simulate the bulk behavior of the micrometer‐sized experimental sample and minimize finite‐size edge artifacts.

We have introduced three key contributors of skyrmion‐to‐stripe transformation to the Landau–Lifshitz–Gilbert (LLG) equation to investigate the magnetization dynamics: SOT, STT, and finite temperature thermal fluctuations.

1. SOT: Current injection in the Pt/FGaT heterostructure generates an interfacial SOT. This was implemented by adding damping‐like and field‐like torque terms to the LLG equation
(1) $$\tau_{SOT}=-\frac{\gamma_{0}}{1+\alpha^{2}}a_{J}\left(\left(1+\alpha \xi \right)m\times \left(m\times \;p\right)+\left(\xi -\alpha \right)m\times p\right)$$

Here *p* = (0, 1, 0) represents the polarization direction of the spin current, which is transverse to the electrical current direction, *θ*_SH_ = 0.22 is the spin‐Hall angle [47], *J* is the electrical current density, d is the layer thickness, and *a*_J_ represents the magnitude of the damping‐like torque, given by

(2) $$a_{J}=\frac{\hbar \cdot \theta_{SH}\cdot J}{2e\cdot d\cdot M_{sat}}$$

The dimensionless parameter *x*_i_ = *b*_J_/*a*_J_ quantifies the ratio between field‐like and damping‐like torques, a small ratio of *x*_i_ = 0.08 was considered in simulations and is consistent with experimental characterization.

1. STT: Since the current flows in‐plane through the FGaT layer, the Spin‐Transfer Torque was modeled using the Zhang‐Li formalism, which describes the interaction between the polarized current and the local magnetization gradient. This contribution accounts for the adiabatic and non‐adiabatic torques, and a polarization ratio Pol = 0.415 was used in simulations [65].
2. Thermal Effects: Finite temperature effects were included to model the thermodynamic stability and phase transitions of the magnetic textures. Temperature‐dependent material parameters were scaled with phenomenological scaling laws with *M*_sat_(T) as follows:
(3) $$A_{ex}\propto Msat{\left(T\right)}^{2},K_{u1}\propto Msat{\left(T\right)}^{3},D\propto Msat{\left(T\right)}^{2}\;$$

Combining with the stochastic thermal field, this ensures that the simulation captures the entropy‐driven mechanisms facilitating the escape from the metastable skyrmion state.

### 5.5 Electrical Transport Measurements

All current pulses were supplied by a Keithley 6221 current source. For the AHR measurements, a writing pulse of a specific amplitude and duration was first applied. A delay of 10 s was introduced after each write pulse to allow for thermal dissipation and minimize any residual Joule heating effects. Following the delay, a low‐frequency (18.3 Hz) AC read current was applied, and the resulting Hall voltage was measured using a Stanford Research Systems SR830 lock‐in amplifier. To improve the signal‐to‐noise ratio, each data point reported represents the average of three independent readings.

### 5.6 QANN Simulation

The QANN is a Multi‐Layer Perceptron (MLP) consisting of an input layer with 784 neurons, a 128‐dimensional hidden layer, and a 10‐dimensional output layer. For each layer, the quantization threshold was dynamically calculated by multiplying the mean of the absolute values of the full‐precision weights by a scaling factor (0.7 in our method). Any weight greater than the positive threshold was mapped to 1, any weight less than the negative threshold was mapped to ‐1, and all other weights were set to 0. A ReLU function was used for activation after the hidden layer, while the output layer utilizes a Softmax function to generate probabilities for the ten classes. The predicted label corresponds to the class with the highest probability. The network processes 28 × 28 pixel images from a dataset with a standard split of 60 000 training and 10 000 testing images. Training was conducted with a batch size of 300.

## Conflicts of Interest

The authors declare no conflicts of interest.

## Supporting information




**Supporting File 1**: adma73438‐sup‐0001‐SuppMat.docx.

**Supporting File 2**: adma73438‐sup‐0002‐FigureS1‐S18.zip.

**Supporting File 3**: adma73438‐sup‐0003‐VideoS1.mp4.

## Data Availability

The data that support the findings of this study are available from the corresponding author upon reasonable request.
