# Supplementary material for: Room‐Temperature Skyrmionic Synapse in 2D Ferromagnet Fe3GaTe2 Operating via Collective Spin Texture Transformation
Source: Adv Mater. 2026 May 20;38(49):e20288. doi: 10.1002/adma.202520288 (PMC13532421; doi:10.1002/adma.202520288)
Supplement: Supplementary file 1 [file ADMA-38-e20288-s001.docx]

**Supporting Information**

**Room**-**Temperature Skyrmionic Synapse in 2D Ferromagnet Fe_3_GaTe_2_ Operating via Collective Spin Texture Transformation**

*Jixiang Huang, Tongji Zhu, PeiYu Cai, Xiaoming Ma, Zhen Wang, Ruifu Zhang, Yi Hao, Jingdi Lu, Liubing He, Jun Xu, Yuejie Zhang, Wanjun Jiang, Jing Tao, Shiming Lei, Pavel Parchinskiy, Jing Teng,* Lingfei Wang,* Wei Niu,* Elton J.G. Santos,* Xiaoqian Zhang,* Peng Li**

1. **Thickness Dependence of Magnetic Domains and Skyrmions**

The formation and stability of magnetic domains and skyrmions in Fe_3_GaTe_2_ (FGaT) are highly dependent on the flake thickness. Our magnetic force microscopy (MFM) investigations (**Figure** **S1** a-e) reveal a clear evolutionary pathway of the magnetic domain configuration as the flake thickness increases. In the thinnest flakes (e.g., <30 nm), strong perpendicular magnetic anisotropy (PMA) dominates, favoring a uniform, single-domain magnetic state. As the thickness increases, magnetostatic (dipolar interaction) energy begins to counteract the PMA, leading to the nucleation of disordered stripe domains. With a further increase in thickness, these stripes transform into an ordered, labyrinthine pattern.^[1]^ Crucially, only those areas with ordered labyrinthine domains can be transformed into a stable skyrmion lattice via a field-cooling (FC) protocol while the single-domain and disordered stripe states do not support this transformation.

This thickness-driven evolution is quantitatively captured in the non-monotonic dependence of the domain width and skyrmion diameter on flake thickness (Figure S1 f). In the thinner regime (approximately 30-90 nm), the domain width and skyrmion size are observed to decrease as thickness increases. This behavior is attributed to the dominant role of PMA relative to the dipolar energy in this range. Conversely, for thicker flakes (>90 nm), the influence of the long-range magnetostatic dipolar energy becomes more pronounced, causing the domain width to increase with thickness, a trend consistent with the predictions of Kittel’s law for stripe domains.^[2-3]^ Therefore, while the Dzyaloshinskii-Moriya interaction (DMI) provides the essential chirality for forming skyrmions, their ultimate magnetic structures are dictated by this thickness-dependent competition between PMA, DMI and dipolar interactions.

**Figure S1.** a-e) MFM images of FGaT flakes with different thickness before and after FC, respectively. f) skyrmion diameter (blue dots) and domain width (red dots) as a function of FGaT flake thickness.

2. **Influence of Surface Oxidation on Magnetic Domain Structure**

To elucidate the role of the oxidation-induced O-FGaT/FGaT interface on domain stability and skyrmion formation, we systematically tracked the evolution of magnetic domains in flakes of varying thicknesses via MFM after exposure to air for different durations, as shown in **Figure** **S2**. The results reveal a clear degradation of the magnetic domain structure over time, with the effect being most pronounced in thinner samples. The 50 nm flake, which initially exhibits the ordered labyrinthine domains necessary for skyrmion formation, transitions to disordered stripes after 2 hours and reverts completely to a single-domain state after 24 hours. A similar degradation from ordered to disordered stripes is observed in the 80 nm flake, while the thickest 180 nm flake remains largely unaffected over the same period. To understand the underlying physical mechanism, we performed high-angle annular dark-field scanning transmission electron microscopy (HAADF-STEM) measurements on a flake after 1 hour of air exposure (**Figure S3**). The images confirm the formation of an amorphous surface oxide layer, and energy-dispersive X-ray spectroscopy (EDX) indicates that oxygen atoms incorporate into the lattice, primarily substituting Te sites.^[4]^

Taken together, these findings strongly suggest that surface oxidation is detrimental to the stability of the ordered magnetic textures required for skyrmion formation. The process can be interpreted as a reduction of the flake’s effective thickness, causing the domain configuration to degenerate to a state characteristic of a thinner, pristine flake. This supports the conclusion that the DMI in the FGaT system is intrinsic to its bulk crystal structure, rather than an effect induced at an oxide interface.

**Figure S2.** MFM images correspond to fresh samples, samples oxidized at 120 °C for 2 hours, and samples oxidized at room temperature (RT) for 24 hours, with thicknesses of a-c) 50 nm, d-f) 80 nm, and g-i) 180 nm, respectively.

**Figure S3.** a) HAADF-STEM images of FGaT samples after oxidation. b-e) EDX maps of the region with the oxidized surface layer.

3. **Field-Dependent Evolution of the labyrinthine Stripe Domain**

For comparison with the evolution of the skyrmion state, we also investigated the magnetic field response of a flake initialized in the labyrinthine-domain state at room temperature. As shown in **Figure** **S4**, we used MFM to track the domain evolution during a full magnetic field sweep. Starting from magnetic saturation (e.g., at +470 G), stripe domains nucleate and grow as the field is reduced towards zero. As the field is subsequently reversed, the domains are progressively annihilated until the sample reaches saturation in the opposite direction.

This process exhibits a clear, reproducible hysteresis, which is consistent with the shape of the hysteresis loops shown in the center of Figure S4. Crucially, at no point during this field sweep protocol was a transition between the labyrinthine-domain state and a skyrmion state observed. This confirms that the formation of a stable skyrmion lattice is not a spontaneous process but requires a specific FC procedure described in the main text.

**Figure S4.** Magneto-Optical Kerr Effect (MOKE) hysteresis loop at room temperature and corresponding MFM images at each magnetic field marked in the loop, the scanning sequence is shown as the number. Scale bar:1μm.

4. **Current-driven skyrmion-stripe transition**

**Figure** **S5** presents the complete series of MFM images for the current-driven domain evolution experiment summarized in Figure 2 of the main text. Panels (a-f) show the step-by-step evolution of the magnetic texture under a train of positive current pulses. Panels (g-j) show the corresponding evolution under a train of negative current pulses. The results indicate that under positive current pulses, skyrmions in FGaT are gradually elongated into stripe domains. Driven by the current, the domain walls propagate inward, compressing and subsequently annihilating the skyrmions along their path. Conversely, when the polarity of the injected current is reversed, the stripe domains do not revert to the skyrmion state; instead, only the direction of domain wall propagation is reversed. This demonstrates that the current-driven skyrmion-to-stripe domain transition is an irreversible process.

**Figure S5.** a-f) FGaT skyrmion evolution under positive current pulses. g-j) FGaT skyrmion evolution under negative current pulses. The corresponding current density is indicated by the arrow below, unit:10^6^A/cm^2^, Scale bar: 2 μm.

To further substantiate the evolution of the magnetic domains, we tracked individual stripe-domain segments frame by frame. As shown in main text Figure. 2b-c, applying positive current pulses causes the skyrmions located near the edge of the current path to elongate and gradually transform into stripe-like textures. With continued pulsing, the domain walls of these stripes propagate inward. This propagation, highlighted by the red dashed lines, exerts increasing pressure on the skyrmions situated inside the current path, marked as the white dots, leading to their progressive compression and eventual annihilation. These observations confirm that each skyrmion continuously shrinks and collapses at nearly its original position, rather than drifting toward or exiting the field-of-view boundary.

In order to achieve an electrically driven transformation from the stripe-domain state back to a skyrmion state, we performed an exploratory study. As shown in **Figure S6**, we applied 1,000 bipolar current pulses (20 µs width, 1.7 × 10^7^ A/cm² current density) in the presence of a constant 250 G out-of-plane (OOP) magnetic field.^[5]^ The MFM images demonstrate that this protocol is partially successful, converting a majority of the stripe domains into skyrmions. We consider that the incomplete conversion is due to inhomogeneities of the mechanically exfoliated FGaT flake, which can create local variations in the energy landscape. The underlying mechanism is likely analogous to the FC protocol. The rapid and localized Joule heating from the bipolar pulses provides the thermal energy for the system to overcome energy barriers and relax into the skyrmion state, which is favored by the external bias field. This result is a crucial proof-of-concept, establishing a viable pathway toward achieving all-electrical, reversible control of skyrmion and stripe-domain states in FGaT devices.

**Figure S6.** a-b) MFM images of the FGaT flake in its (a) initial stripe-domain state, and (b) after the application of 1,000 bipolar current pulses. The scale bar is 1 μm.

5. **Micromagnetic simulation**

To further illustrate the driven mechanism of the current-driven spin texture transformation of FGaT system, we analyze the whole process via micromagnetic simulation based on Mumax3.

5.1. Skyrmion Evolution and Topological Charge Dynamics

Upon applying an in-plane current pulse J ~ 7×10^6^ A/cm^2^ for 40 ns and simultaneously removing the external stabilizing field, we observed a distinct topological phase transition. As shown in the simulation snapshots (**Figure S7**), the isolated skyrmions initially distort along the direction of the SOT-induced effective field. As the pulse continues, the skyrmions elongate and merge, eventually evolving into a labyrinthine stripe domain state. This morphological evolution is in excellent agreement with the experimental observation where circular skyrmions unzip into worm-like textures under current pulses.

**Figure S7.** Snapshots from micromagnetic simulations captured at a) 0 ns, b) 10 ns, c) 10.1 ns, d) 20 ns, e) 30 ns, f) 40 ns, and g) 50 ns. The interval 0-10 ns corresponds to the FC process, followed by current injection starting at 10 ns.

To quantitatively confirm that this transformation involves true skyrmion annihilation rather than displacement (shifting) of magnetic textures out of the device area, we monitored the total topological charge Q of the system over time. **Figure S8** shows the total topological charge exhibits a continuous decay from an initial value of Q ~ −50 (representing a dense skyrmion lattice) to a residual value of Q ~ −5. This >90% reduction in topological charge confirms a massive annihilation event occurring within the material. The residual non-zero charge is attributed to topological remnants and defects, such as Y-junctions and domain wall ends, which are inherent to the disordered stripe state. This result definitively rules out the possibility that skyrmions are merely pushed out of the field of view by the SOT, confirming a genuine topological phase transition.

**Figure S8.** Variation of the topological charge during the current-driven spin texture transformation in FGaT.

5.2. Energetic Origin of the Transformation

We have plotted different energy contributions over the transformation process to investigate the distinct roles of the energy terms (**Figure S9**). It is important to note that a perfect skyrmion lattice at 0 K possesses high topological stability; under applied current, such a rigid lattice would typically exhibit coherent motion rather than deformation. Our simulations reveal that the inclusion of thermal effects, implemented via phenomenological parameter scaling and a stochastic thermal field, was critical to reproducing the experimental phase transition. These thermal fluctuations increase the system's free energy and lower the effective activation barriers, destabilizing the metastable skyrmion lattice and facilitating the topology-changing transformation.

Demagnetization Energy (Dipolar Interaction): Following the removal of the external magnetic field at t = 10 ns, the Zeeman energy vanishes. In this zero-field regime, the skyrmion lattice becomes metastable. The demagnetization energy is observed to decrease continuously during the transformation. This indicates that the labyrinthine stripe state, which minimizes stray fields by establishing equal areas of "Up" and "Down" magnetization, acts as the thermodynamic ground state. Therefore, the demagnetization energy acts as a stabilizing contributor to the system.

Exchange and Anisotropy Energy: Conversely, the internal interaction energies increase during the transition. The elongation of skyrmions into stripes significantly increases the total domain wall length. This imposes an energy penalty visible in the exchange energy term (which in our simulation includes both Heisenberg and DMI contributions). While DMI favors twisting, the massive increase in total domain wall area during the "unzipping" process results in a net increase in this combined energy term. Furthermore, the SOT exerts a torque that tilts the magnetization away from the easy axis, increasing the anisotropy energy.

Active Energy Injection: The total energy plot shows a net increase during the current pulse. This confirms that the SOT and STT currents actively inject energy into the system, providing the necessary work to overcome the topological protection barrier and the local exchange/anisotropy energy barriers.

Therefore, the mechanism can be described as an energy-injection-assisted relaxation: the current pulse pushes the system over the topological barrier, allowing it to relax "downhill" in the Demagnetization energy landscape into the stable stripe phase.

**Figure S9.** Variation of individual energy terms extracted from micromagnetic simulations during the transition from a skyrmion lattice to stripe domains in FGaT. a) total energy, b) exchange energy, c) demagnetization energy, d) anisotropy energy, e) thermal energy, f) Zeeman energy.

5.3. Magnetization Dynamics and Retention

Our simulations show that *M*_z_ decays from a saturated value (*M*_z_/*M*_sat_ ∼ 0.45) in the skyrmion state to nearly zero in the final stripe state. During the 0-10 ns interval, the system evolves into a skyrmion lattice state via a FC process. Subsequently, at t = 10 ns, the external magnetic field is removed, and a driving current is applied. Upon field removal, *M*_z_ exhibits a rapid drop before quickly stabilizing at a new state. Following this, driven by the current, the system gradually transitions from the skyrmion lattice to a stripe domain configuration. The evolution of *M*_z_ throughout this process is illustrated in the inset of **Figure S10.** This vanishing net magnetization (*M*_z_ ~ 0) confirms that the final state is a fully demagnetized labyrinth with equal populations of up and down domains.

**Figure S10.** Time evolution of the out-of-plane magnetization (*M*_z_). The inset displays the variation during the 20-50 ns interval.

Finally, we addressed the reversibility of the transformation, a critical factor for device architecture. Simulation tests applying a reverse current to the relaxed stripe state failed to restore the original skyrmion lattice, as shown in **Figure S11**, which is consistent with the experimental observations. The stripe domains exhibited high retention, remaining stable even after the removal of all external stimuli for extended relaxation times.

**Figure S11.** Evolution of stripe domains under reverse current pulses. The images were captured at a) 0 ns, b) 10 ns, c) 20 ns and d) 30 ns. The current density is 7 × 10^6^A/cm^2^.

This irreversibility is consistent with the drastically reduced net topological charge observed in the final state. While the skyrmion lattice carries a high topological charge, the stripe state is topologically trivial. Re-nucleating the dense topological charge required to restore the lattice from this trivial background imposes a massive nucleation barrier that cannot be overcome by simple current reversal. Furthermore, as indicated in Figure S9, the skyrmion lattice state represents a metastable state characterized by higher demagnetization energy. Consequently, the transition from stripe domains back to skyrmions constitutes an energetically unfavorable inverse thermodynamic process,^[6]^ and thus cannot occur under the drive of a reverse current. This confirms that the device operates as a non-volatile memory, where the "Write" operation is electrical, while the "Erase" operation requires a global thermal reset to near or above *T*_C_.

6. **Electrical Readout of the Skyrmion-to-Stripe Transformation**

6.1 current-driven labyrinth domain evolution

**Figure** **S12** provides the MFM images corresponding to the zero-field-cooled (ZFC) control experiment discussed in Figure 3e of the main text. After initializing the device into a stable labyrinth domain state, a train of current pulses was applied. While the MFM images reveal a significant rearrangement of the stripe domain structure under the influence of the current, this reconfiguration does not produce a corresponding change in the anomalous Hall resistance (AHR). This demonstrates that only the topological transformation from skyrmions to stripes yields a significant electrical response.

**Figure S12.** a-d) magnetic domain evolution with applied current pulses of device 2 after ZFC. The corresponding current density is indicated by the arrow below, unit:10^6^A/cm^2^. The insets in (a) is a photo of the device under test, where the red squares indicate the region of interest in (a-d). The scale bar is 2 μm.

6.2 Reproducibility of AHR response

In this work, we fabricated a total of six heterostructure devices with varying dimensions for MFM and electrical transport characterization. The specific geometric parameters are listed in **Table S1**, and the corresponding Atomic Force Microscopy (AFM) images are presented in **Figure S13**.

**Table S1**. Geometric dimensions of Devices 1-6.

|  | **Device 1** | **Device 2** | **Device 3** | **Device 4** | **Device 5** | **Device 6** |
| --- | --- | --- | --- | --- | --- | --- |
| L*W(μm^2^) | 30*5 | 25*5 | 20*5 | 40*10 | 30*5 | 30*3 |
| thickness(nm) | 48 | 78 | 71 | 82 | 65 | 42 |

**Figure S13.** a-f) AFM images and sample thickness characterization of all the devices used in this work.

Subsequently, we demonstrate the complete AHR response of Device 3 in **Figure S14**. In the low current density regime, the skyrmion lattice remains stable, resulting in negligible changes in the Hall resistance. As the current density increases, the skyrmions gradually begin to elongate into stripe domains and propagate toward the device interior, inducing the annihilation of internal skyrmions. During this process, the AHR decreases gradually, which is consistent with the reduction in *M*_z_ shown in the inset of Figure S10.

With continuous current pulse injection, the magnetic state of the FGaT fully transforms into stripe domains; beyond this point, further current application yields no significant variation in AHR. Consequently, the entire evolution can be categorized into three distinct regimes: the creep regime, the flow regime, and the saturation regime. Notably, within the flow regime, the AHR exhibits a near-linear variation with the number of applied pulses, and the slope can be effectively modulated by the pulse width. Leveraging this linear regime, we constructed a weight-tunable skyrmionic artificial synaptic device.

**Figure** **S14.** a) Anomalous Hall effect (AHE) loop of device 3 with a thickness of 71nm. b) The variation value of Hall resistance(Δ*R*_xy_) as a function of applied current pulses, the red and blue dots represent for FC and ZFC conditions, respectively. c) Variation of Δ*R*_xy_ as a function of the number of applied pulses for different pulse widths.

Furthermore, we validated the reproducibility of this response using Devices 4-6. As illustrated in **Figure S15**, we characterized the AHR evolution as a function of injected pulses for devices with varying dimensions. The results demonstrate that the current-driven spin texture transformation and its corresponding electrical readout can be consistently realized across devices of different sizes. It is worth noting that, to prevent potential device breakdown during testing, the measurements for Devices 4-6 were restricted to the creep and flow regimes.

**Figure S15.** AHR measurements for Device 4-6. a-c) Dependence of the AHR change (Δ*R*_xy_) on current pulse amplitude for various pulse widths, showing the creep and flow regimes. d-f) Accumulated change in Δ*R*_xy_ as a function of the number of applied pulses in the flow regime.

6.3 Endurance and retention of FGaT skyrmionic synapse

Subsequently, we verified the reproducibility of the synaptic functionality. As illustrated in **Figure S16**, we performed multi-cycle write-erase tests on Devices 4 and 5. The results demonstrate that a linear AHR response with respect to the pulse count is maintained across multiple cycles, with the slope remaining substantially consistent. Given that this slope dictates the synaptic weight update, this consistency validates the endurance of our skyrmionic synaptic devices.

Notably, the fifth and sixth cycles were conducted 5 and 10 days after the initial measurement, respectively. During these intervals, the devices were stored under ambient conditions (room temperature and air). The consistent performance after these delays confirms the robust retention capabilities of the proposed devices.

**Figure S16.** Multi-cycle write-erase tests for a) Device 4 and b) Device 5. The fifth and sixth cycles were conducted 5 and 10 days after the initial test, respectively.

7. **Energy Consumption Analysis of FGaT Skyrmion Synapse**

This section details the calculation of the energy consumption for a single synaptic operation in our experimental device and outlines the assumptions used for the scaling projection presented in the main text. The calculation is based on our Hall bar heterostructure device, which consists of a 10 nm Pt layer and a 75 nm FGaT flake, with an active area of 25 × 5 µm^2^. Using a parallel resistor model based on the known resistivities of Pt and FGaT, the total resistance (R) of the heterostructure is calculated to be 35.9 Ω.

The energy consumption (W) for a single synaptic operation is determined by the Joule heating from a single current pulse, given by the formula:

$$\begin{aligned} W=I^{2}\cdot R\cdot t_{pulse} \#\left( 1 \right) \end{aligned}$$

For the experimental parameters used in this study, with a pulse amplitude (I) of 7 mA and pulse widths (t_pulse_) ranging from 0.2 ms to 1 ms, the single-operation energy consumption is calculated to be in the range of 351.8 to 1759.1 nJ.

Then, we consider the projection of W by device geometry scaling, the current amplitude of injected pulses is calculated with:

$$\begin{aligned} I=J\cdot w\cdot t \#\left( 2 \right) \end{aligned}$$

where J, w, and t represent current density, device width, and thickness, respectively. Meanwhile,

$$\begin{aligned} R=\frac{\rho\cdot l}{w\cdot t} \#\left( 3 \right) \end{aligned}$$

is the resistance of the active region. Here, ρ represents for the total resistivity of the heterojunction device. The pulse width, t_pulse_, corresponds to the time required to drive the skyrmion-to-stripe transformation. Assuming a constant driven current density and domain wall velocity, t_pulse_ is directly proportional to the length of the active region.

Deriving from these equations, we find that the energy consumption scales as:

$$\begin{aligned} W\propto l^{2}\cdot w\#\left( 4 \right) \end{aligned}$$

Consequently, we project that by scaling the device dimensions down to 180 nm × 180 nm, the energy per operation can be reduced to approximately 0.66 pJ. This projected efficiency is comparable to state-of-the-art synaptic devices based on Resistive random-access memory (RRAM) and Phase-change memory (PCM).

Furthermore, we calculated the energy consumption per single operation for Devices 4-6, plotted it as a function of device dimension (e.g., channel width), and compared it with the theoretical predictions discussed above. As shown in **Figure S17**, the solid stars (representing experimental data for Devices 4-6) exhibit excellent agreement with the blue dashed line. This result validates the rationality of our energy consumption projections based on dimensional scaling.

**Figure S17**. Comparison of the measured and predicted energy consumption per single synaptic operation for Devices 4-6. The solid stars represent the experimental data, while the blue dashed line indicates the theoretical prediction.

8. **Performance Benchmark Against CMOS-Based Synapses**

Next, we compare the performance of our FGaT skyrmion artificial synaptic device with two conventional CMOS-based synapse technologies.^[7-8]^ To ensure a direct and fair comparison, all devices are scaled to 180 nm technology node, and the performance of a single synapse is analyzed. As mentioned in the main text, our proposed device utilizes a magnetic tunnel junction (MTJ) for readout, requiring one transistor and one MTJ (1T1M) per unit.^[9]^

As shown in **Figure** **S18**a, our device offers a substantial advantage in integration density. The area of our synaptic unit is projected to be approximately 10 times smaller than a standard 6T SRAM-based synapse and 78 times smaller than a 1T1C (one transistor, one capacitor) capacitor-based synapse. Furthermore, our single device provides three distinct weight states and has the potential for more in the future, which could offer even greater advantages in large-scale arrays where high state density is crucial.

In terms of energy consumption, our device's non-volatile weight storage provides a significant benefit. As shown in Figure S18b, the single-operation energy consumption of our FGaT skyrmion synapse is projected to be over 9 times lower than that of the capacitor synapse and more than 5100 times lower than the SRAM synapse (the detailed data are listed in **Table** **S2**). This comprehensive analysis demonstrates that artificial synapses based on the FGaT skyrmion-to-stripe transformation are highly competitive, showing superior performance in integration density and power efficiency compared to traditional CMOS digital synapse implementations. This highlights their strong potential for future neuromorphic systems.

**Figure S18.** a) Comparison of the projected physical area for a single-bit synaptic unit for the FGaT skyrmion device, a 6T-SRAM cell, and a capacitor-based synapse. b) Comparison of the projected energy consumption for a single synaptic operation between the three device types. All values are calculated or projected for a 180 nm technology node for a direct comparison.

Table S2. Performance Benchmark of the FGaT Skyrmion Synapse Against CMOS Synapses

|  | FGaT skyrmion  synapse | CMOS synapses | |
| --- | --- | --- | --- |
|  |  | Capacitor | SRAM |
| Area(μm^2^) | 0.26 | 20.26 | 2.6 |
| Weight number | 3 | 2 | 2 |
| Device number | 2(1T1M) | 2(T) | 6(T) |
| Energy cost/operation (pJ) | 0.66 | 6 | 3400 |
| Non-volatile | Y | N | N |

**Reference:**

[1]	H. Zhang, Y.-T. Shao, X. Chen, B. Zhang, T. Wang, F. Meng, K. Xu, P. Meisenheimer, X. Chen, X. Huang, *Nat. Commun.* **2024**, 15, 3828.

[2]	S. Jin, Y. Wang, H. Zheng, S. Dong, K. Han, Z. Wang, G. Wang, X. Jiang, X. Wang, J. Hong, H. Huang, Y. Zhang, T. L. Xia, X. Wang, *Nano. Lett.* **2024**, 24, 5467.

[3]	H. Zhao, C. Yang, Y. Liu, Q. Wang, Y. Wu, Q. Mu, F. Hou, T. Min, T. Li, *Adv. Mater.* **2025**, e2500534.

[4]	Y. Li, X. Hu, A. Fereidouni, R. Basnet, K. Pandey, J. Wen, Y. Liu, H. Zheng, H. O. H. Churchill, J. Hu, A. K. Petford-Long, C. Phatak, *ACS Appl. Nano. Mater.* **2023**, 6, 4390.

[5]	T.-E. Park, L. Peng, J. Liang, A. Hallal, F. S. Yasin, X. Zhang, K. M. Song, S. J. Kim, K. Kim, M. Weigand, G. Schütz, S. Finizio, J. Raabe, K. Garcia, J. Xia, Y. Zhou, M. Ezawa, X. Liu, J. Chang, H. C. Koo, Y. D. Kim, M. Chshiev, A. Fert, H. Yang, X. Yu, S. Woo, *Phys. Rev. B* **2021**, 103, 104410

[6] N. Nagaosa, Y. Tokura, Nat. Nanotechnol. 2013, 8, 899.

[7]	S. Höppner, Y. Yan, A. Dixius, S. Scholze, J. Partzsch, M. Stolba, F. Kelber, B. Vogginger, F. Neumärker, G. Ellguth, *arXiv:2103.08392* **2021**.

[8]	R. Oshio, T. Kuwahara, T. Aoki, M. Kimura, Y. Nakashima, *Sci. Rep.* **2025**, 15, 3954.

[9]	T. da Câmara Santa Clara Gomes, Y. Sassi, D. Sanz-Hernández, S. Krishnia, S. Collin, M.-B. Martin, P. Seneor, V. Cros, J. Grollier, N. Reyren, *Nat. Electron.* **2025**, 8, 204.
